# Electrochemical Detection of Plasma Immunoglobulin as a Biomarker for Alzheimer’s Disease

**DOI:** 10.3390/s17112464

**Published:** 2017-10-27

**Authors:** Goulielmos-Zois Garyfallou, Orlando Ketebu, Samet Şahin, Elizabeta B. Mukaetova-Ladinska, Michael Catt, Eileen Hao Yu

**Affiliations:** 1 School of Chemical Engineering and Advance Materials, Newcastle University, Newcastle upon Tyne NE1 7RU, UK; william.garyfallou@quantumdx.com (G.-Z.G.); orlandok2@yahoo.com (O.K.); sametsahin@me.com (S.Ş.); 2 Department of Chemical and Process Engineering, Faculty of Engineering, Bilecik Şeyh Edebali University, 11230 Bilecik, Turkey; 3 Department of Neuroscience, Psychology and Behaviour, University of Leicester, University Road, Leicester LE1 7RH, UK; eml12@le.ac.uk; 4 Institute of Neuroscience, Faculty of Medical Sciences, Newcastle University, Newcastle upon Tyne NE2 4HH, UK; michael.catt@ncl.ac.uk

**Keywords:** Alzheimer’s disease, electrochemical impedance spectroscopy, plasma immunoglobulin, polyclonal rabbit Anti-human immunoglobulin, direct immunosensor

## Abstract

The clinical diagnosis and treatment of Alzheimer’s disease (AD) represent a challenge to clinicians due to the variability of clinical symptomatology as well as the unavailability of reliable diagnostic tests. In this study, the development of a novel electrochemical assay and its potential to detect peripheral blood biomarkers to diagnose AD using plasma immunoglobulins is investigated. The immunosensor employs a gold electrode as the immobilizing substrate, albumin depleted plasma immunoglobulin as the biomarker, and polyclonal rabbit Anti-human immunoglobulin (against IgA, IgG, IgM) as the receptor for plasma conjugation. The assay showed good response, sensitivity and reproducibility in differentiating plasma immunoglobulin from AD and control subjects down to 10^−9^ dilutions of plasma immunoglobulin representing plasma content concentrations in the pg mL^−1^ range. The newly developed assay is highly sensitive, less time consuming, easy to handle, can be easily modified to detect other dementia-related biomarkers in blood samples, and can be easily integrated into portable devices.

## 1. Introduction

Diagnostic tests capable of detecting dementia syndromes with high sensitivity and specificity in clinical settings are urgently needed. The need for diagnostic methods to detect dementia is becoming more acute with the increasing numbers worldwide of older people who may also suffer from chronic co-morbid illnesses and who are at high risk of developing memory problems. At present, there are at least 36 million people living with dementia worldwide, with the prevalence rates estimated to double every 20 years [1]. This represents a rapidly growing challenge for both the health and social care systems, with implications for economic plans and the development of adequate clinical and support services to diagnose, treat, house, maintain in the community, and deliver continuous care for this population. The enormous potential available for identifying a minimally-invasive, easily-accessible blood measure as an effective Alzheimer’s disease (AD) biomarker currently remains unfulfilled [2]. 

With a rapidly growing ageing population and dementia prevalence rates doubling in the following decade as reported by Alzheimer’s Society UK, it has become imperative to provide general practitioners with novel diagnostic tools aimed at both early diagnosis and care for AD patients. The currently suggested toolkit includes a range of cognitive assessment scales (including the 6-item Cognitive Impairment Test, MiniCog and the Functional Activities Questionnaire, the Informant Questionnaire on Cognitive Decline in the Elderly (IQCODE), the General Practitioner Assessment of Cognition (GPCOG) and Mini Mental State Exam (MMSE)). However, the sensitivity and specificity of the aforementioned tests for dementia detection can vary greatly and they are therefore meant to be used as signposts rather than to provide a comprehensive clinical evaluation of the dementia syndrome [3]. In order to minimize unnecessary interventions, prevent physical and mental health crises, and make best use of available resources, it is imperative to develop an objective tool that will improve the understanding of the central brain dementia process, enhance the early diagnosis of dementia, facilitate early initiation of anti-dementia therapies and monitor therapeutic outcome(s). One of these objective tools is the development of highly sensitive and specific peripheral (blood) biomarkers for diagnosing dementia.

The development of peripheral biomarkers for dementia is based on blood products identified either in proteomic studies or those using traditional hallmarks for dementia. Proteomic studies have identified a number of potential peripheral (blood) biomarkers for dementia [4,5] including markers for: inflammation [6,7], apoptosis [8], immune response [6,9,10], signalling proteins [11], neuronal energy deficiency [12] and systemic redox regulation [13]. Some of these highest quality biomarker candidates have been subjected to an iterative process of verification and assay development: plasma α-2-macroglobulin and complement factor H, although elevated in AD subjects, compared to controls had low sensitivity (39–49%), though high specificity (80%, [6]), whereas plasma clusterin (amyloid chaperone protein) increase in AD subjects has yet to be confirmed in other studies [8,14]. 

Recent proteomic studies support the use of multianalyte profiling, e.g. subsets of 4–5 plasma proteins, either closely related to the amyloid protein precursor (APP) and tau protein [15] and inflammation [16], or lipids associated with cell membrane integrity [17] for diagnosing and even predicting dementia. This suggests that a combination of several biomarkers may be best suited for developing a blood test for dementia. This approach calls for development of novel methodologies that can accommodate and enhance the detection of such an array of dementia peripheral biomarkers.

In contrast to the ELISA immunoassays, which have multiple variables that can influence their performance (avidity and concentration of capture and detection antibodies, incubation time and temperature, sample volume and dilution, enzyme and substrate types, quality of the detector) [4], biosensor assays consist of three components—a receptor (an antibody or binding protein), a transducer (for example an electrode) and signal processing electronics. Though electrochemical biosensors may suffer from influences similar to other assays such as temperature, avidity, and time, they offer advantages in the simplicity of fabrication, reduced costs and the potential for fully automated microfabrication approaches which can result in nano-sized devices therefore reducing the amount of sample required by conventional approaches [18]. Biosensor assays have already been used to detect peripheral (serum) markers in neurological diseases, thus paving the way for potential use in dementia studies. Bryan et al. developed a novel approach in utilising α-synuclein modified electrodes to sample the autoantibodies generated in the course of Parkinson’s disease (PD) [19]. This peptide-based electroanalytical assay not only distinguished between PD and control individuals, but also mapped out the disease progression. The principles of this assay can be translated for other circulating autoantibodies against the classical hallmarks of AD previously reported in blood/serum such as tau protein [20] and amyloid beta [21,22]. Similarly, immunoglobulins, as identified in proteomic plasma studies, appear to be increased in dementia [6]. 

Our recent work on blood immunoglobulins, using an indirect ELISA, showed an increase in platelet immunoglobulin content in drug naive subjects with Alzheimer’s disease, which was normalised following treatment with anti-dementia (cholinesterase inhibitor) drugs, whereas the plasma immunoglobulin levels did not discriminate between dementia and control subjects [23]. This suggests that immunoglobulins and circulating autoantibodies have the potential to be used as dementia biomarkers and may reflect the efficacy of the currently available anti-dementia treatments. However, in the light of inconsistencies in the findings of different groups [6,23], it becomes apparent that the methods for detection need to be improved and furthermore be made compatible for use in multiplex platforms. Direct electrochemical detection of plasma proteins may overcome this limitation. 

In the current study, a novel method was developed for the direct detection of plasma immunoglobulins as a biomarker for AD using electrochemical techniques. The assay described herein employed the use of polyclonal rabbit Anti-human immunoglobulin immobilized on the surface of a gold electrode as the receptor element, which was subsequently exposed to a series of 10-fold dilutions ranging from 10^−3^ down to 10^−12^ of depleted plasma solutions. Changes in the surface properties induced by the conjugation event between receptor and the target immunoglobins were detected using cyclic voltammetry and electrochemical impedance spectroscopy. A linear correlation between electrochemical signals against increasing concentrations of plasma samples was obtained and the immunosensor electrode was able to differentiate between plasma immunoglobulin from AD and control subjects.

## 2. Experimental

### 2.1. Materials and Chemicals

Albumin depleted plasma samples from two controls (hereon referred to as cases 2 and 4–78 and 71 years old, respectively) and two AD subjects (hereon referred to as cases 1 and 3–72 and 76 years old, respectively) were used in the current study. The diagnosis of AD was made according to the NINCDS-ADRDA criteria for dementia [24]. The blood samples from both cases were collected as a part of the study on peripheral blood biomarkers. The albumin depleted plasma samples were papered after Mukaetova-Ladinska et al. 2014 [14,23].

Alumina particles (γ-Al_2_O_3_), 0.3 μm and 0.05 μm in diameter, were purchased from CH Instruments (Austin, TX, USA). Potassium ferrocyanide (K_4_[Fe(CN)_6_]·3H_2_O) and potassium ferricyanide (K_3_Fe(CN)_6_) were purchased from Alfa Aesar (Lancashire, UK). Potassium hydrogen phosphate (K_2_HPO_4_), potassium dihydrogen phosphate (KH_2_PO_4_), glutaraldehyde solution, cysteamine dihydrochloride and sulfuric acid (0.1 M H_2_SO_4_, 98%) were purchased from Sigma Aldrich (Dorset, UK). Polyclonal rabbit Pan immunoglobulin (Anti-human IgA, IgG, IgM) antibody (pAb AO19002) was purchased from Dako (Cambridgeshire, UK) and skimmed milk (0.1% milk; marvel) was purchased from Waitrose (Tyne and Wear, UK). Working dilutions were prepared in PBS pH 7. All chemicals were used as received without further purification. Solutions were prepared using Millipore deionized water (~18 MΩ cm). The supporting electrolyte for the electrochemical studies was phosphate buffered saline (PBS) solution 1X (150 mM NaCl, 0.8 mM K_2_HPO_4_ and 0.15 mM KH_2_PO_4_) (0.1 mol L^−1^, Ph = 6.98). The pH measurements were performed using a pH meter with a combined glass electrode from Hanna Instruments HI 83141, (Bedfordshire, UK). Absolute ethanol was purchased from Fischer Scientific (Loughborough, UK).

### 2.2. Immobilization of Polyclonal Rabbit Anti-Human Immunoglobulin and Depleted Plasma Immunoglobulin on Gold (Au) Electrode

Gold electrode (1.6 mm diameter) was polished in 0.05 and 0.3 microns alumina polish powder and rinsed with distilled water. Two-step crosslinking of antibodies was performed as described elsewhere [25]. Briefly, the electrode was subsequently electrochemically cleaned via cyclic voltammetry in 0.1 M H_2_SO_4_ at potentials between 0–1.5 V, at a scan rate 100 mV s^−1^ for 20 cycles and dipped in 200 μL ethanolic solution of 10 mM cysteamine for 3 h in the dark for self-assembly of a monolayer (SAM) of thiol functional groups on the Au surface. The electrode was then drop washed immediately with ethanol, dried using a nitrogen stream and dipped into 200 μL aqueous glutaraldehyde solution (2.5%) for 30 min, followed by a washing step with PBS, drying with a nitrogen stream, and immediately dipping in 200 μL polyclonal rabbit Anti-human immunoglobulin diluted 3:1000 (*v*/*v*, approximately 10 μg ml^−1^) in 2 mL eppendorf tubes, placed in a 50 mL centrifuge tube and stored at 4 °C overnight.

**Figure 1:**
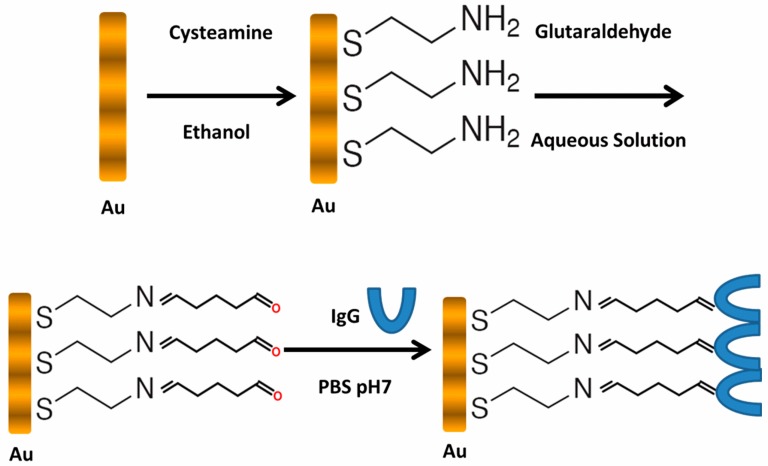
Schematic diagram showing the stepwise modification of gold electrode with cysteamine, glutaraldehyde and polyclonal rabbit Anti-human IgG.

The polyclonal rabbit Anti-human immunoglobulin functionalised electrode was rinsed with PBS pH 7, dipped in 200 μL of 1% milk and stored at room temperature for 1 h. Following a final washing step with PBS, electrochemical measurements were conducted as described in Section 2.3. Initial measurements were carried out on the antibody/milk (AB + Milk) coated surface, followed by exposure to PBS (Baseline) for 15 min. Milk protein was used to block the surface to reduce unspecific binding. Subsequently, the electrode was exposed to 20 μL depleted plasma dilutions (15 min each) by pipetting directly on the surface, starting from the lowest dilution (10^−12^) and leading towards 10^−3^ which was the highest plasma concentration used for these measurements. The total protein contents of the samples used within this study were determined and showed similar protein concentration among the 4 analysed samples, ranging between 35.70 μg mL^−1^ to 36.89 μg mL^−1^. Therefore, the range of dilutions used here includes extremely small concentrations in the sub pg mL^−1^ range (dilutions 10^−12^ to 10^−10^) up to μg mL^−1^ for the more concentrated samples (10^−3^ dilution). Electrochemical measurements were performed for each 10-fold dilution used. Figure 1 shows the schematic diagram for the stepwise modification of gold electrode with plasma immunoglobulin/polyclonal rabbit Anti-human immunoglobulin conjugation. 

### 2.3. Electrochemical Characterization of Modified Electrodes

Cyclic voltammetry (CV) and electrochemical impedance spectroscopy (EIS) were used for the electrode characterization. All electrochemical measurements were carried out using an Autolab PGSTAT302 potentiostat equipped with an FRA module, purchased from EcoChemie (Utrecht, the Netherlands). The CVs were obtained in 10 mM PBS containing 5 mM ferrocyanide/ferricyanide [Fe (CN)_6_]^4−/3−^ and 150 mM NaCl as the supporting electrolyte at a scan rate of 50 mV s^−1^. EIS analyses were carried out in the same solution by determining the open circuit potential (0.22 mV) and applying this as the bias potential, using an AC with 10 mV amplitude in the frequency range 0.1 Hz–10 kHz. A three-electrode cell was used with modified gold electrode as the working electrode, Ag/AgCl (3 M NaCl, 0.208 V vs. SHE) as the reference electrode and a platinum wire as the counter electrode.

## 3. Results and Discussion

### 3.1. Modification of Gold Electrode with Polyclonal Rabbit Anti-Human Ig

**Figure 2:**
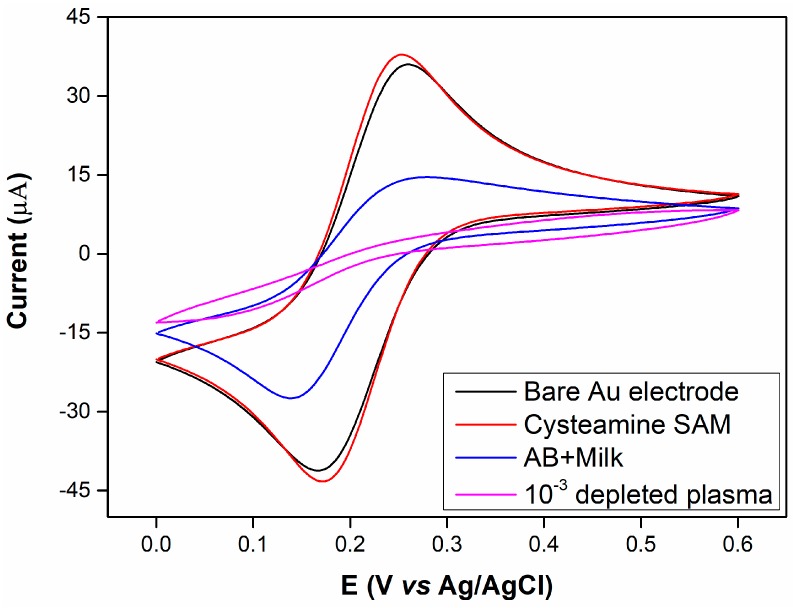
Cyclic voltammetries (CVs) (scan rate: 50 mV s^−1^) of bare Au electrode (black line), Au/Cysteamine-SAM (red line), Au/Cysteamine-SAM /Polyclonal rabbit Anti-human IgG/1% milk (blue line), Au/Cysteamine-SAM /Polyclonal rabbit Anti-human IgG/1% milk /10^−3^ plasma dilution (pink line). Tested in 10 mM PBS containing 5 mM [Fe (CN)_6_]^4−/3−^ and 150 mM NaCl. Electrode surface area: 0.02 cm^2^.

Figure 2 shows the CVs of the gold electrode with stepwise antibody and depleted plasma immunoglobulin assembly. The CVs show well-defined oxidation and reduction peaks for the redox probe ([Fe(CN)_6_]^4−/3−^) observed at the bare gold electrode and cysteamine SAM whereas the peak current decreased after coating the gold electrode surface with polyclonal Ig and a high (10^−3^) plasma concentration. It can be seen that the cysteamine layer did not severely affect electrode behaviour as would be expected but rather the peak current increased and shifted slightly towards more negative voltages. 

The formation of a SAM, and subsequent immobilisation of biomaterials, on the Au surface would be expected to induce decreases in the observed peak currents due to the blocking properties of these materials in comparison with the high conductivity of Au. Figure 2 indicates that the initial cysteamine SAM induced a slight current increase and a small voltage shift towards a more electronegative potential. 

Kerekovic et al. investigated the electrochemical behaviour of cysteamine coated Au electrodes at different pH values in order to determine the effects of surface charge as this is altered due to the isoelectric point of cysteamine [26]. They indicate that at pH 9, most of the amine groups (pKa = 8.27) of cysteamine are neutral and therefore electron transfer is somewhat impeded because of the negative charge of the redox probe. They further show that at pH 3 the peak current for a cysteamine coated Au surface is slightly higher than the bare Au surface and suggest that the reason for this is that amine groups of cysteamine are fully protonated introducing a positive surface charge which attracts rather than repels the negatively charged redox probe.

Close examination of their data further indicates that a small shift towards negative voltages is also observed. This shows similar trend to our observations for our system where at pH 7 it can be expected that the cysteamine layer will be slightly positively charged and therefore attract the negatively charged redox probe, increasing the peak current.

### 3.2. Immobilization of Depleted Plasma Immunoglobulin (Control) and AD Case on Cysteamine-Ig Antibody Coated Gold Electrode

**Figure 3:**
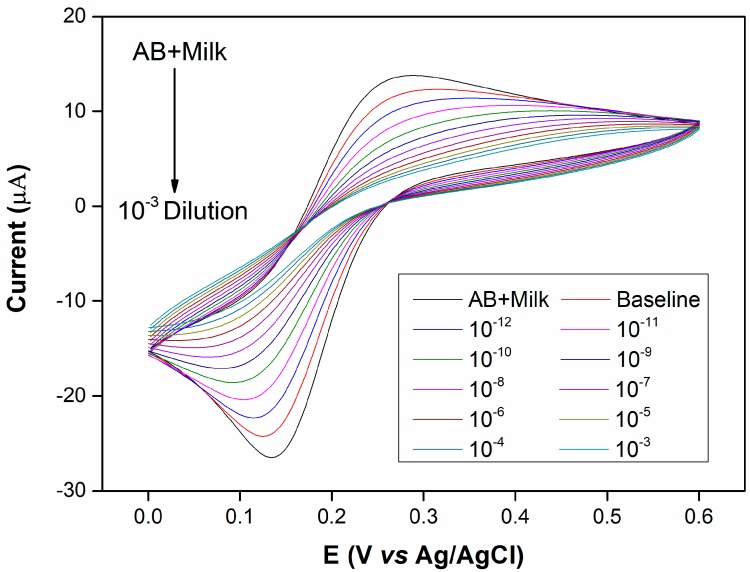
CVs (scan rate: 50 mV s^−1^) carried out for the rabbit anti-human IgG covered surface, baseline solution (plain PBS) and increasing plasma concentrations from 10^−12^ to 10^−3^ 10-fold dilutions of albumin depleted human plasma for the Alzheimer’s disease (AD) (1) case. Tested in 10 mM PBS containing 5 mM [Fe (CN)_6_]^4−/3−^ and 150 mM NaCl. Electrode surface area: 0.02 cm^2^. Control (2-a) results were presented in Figure S1.

**Figure 4:**
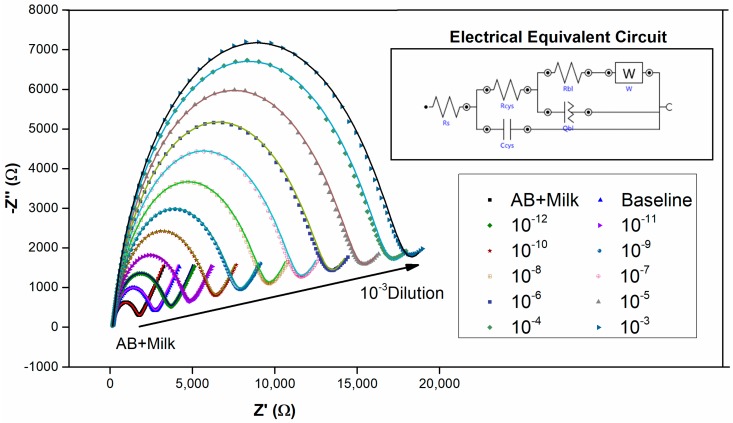
Electrochemical impedance spectroscopy (EIS) measurements carried out at OCP of 0.22 mV for the rabbit anti-human IgG covered surface, baseline solution (plain PBS) and increasing plasma concentrations from 10^−12^ to 10^−3^ 10-fold dilutions of albumin depleted human plasma for the AD (1) case. Inset figure is the electrical equivalent circuit which was used for data fitting and the solid lines represent fitted data. R_s_ is solution resistance, R_cys_ and C_cys_ are the resistance and capacitance of the cysteamine layer, R_bl_ is resistance of the biolayer and Q_bl_ a constant phase element representing the capacitance of the biolayer (*n* > 0.7 for our data) and W is the Warburg impedance representing diffusion. EIS test parameters: AC amplitude: 10 mV, frequency range: 0.1 Hz–10 kHz. Tested in 10 mM PBS containing 5 mM [Fe (CN)_6_]^4−/3−^ and 150 mM NaCl. Electrode surface area: 0.02 cm^2^. Control (2-a) results were presented in Figure S2.

Figure 3 and Figure 4 depict the voltammetric and impedimetric responses for AD case (1) upon the exposure of the rabbit anti-human IgG covered surface to increasing concentrations of human plasma. The subsequent biolayer (AB + Milk) on the Au-cysteamine surface induces a decrease in the observed currents as depicted by voltammetry and an increase in the charge transfer resistance and a decrease in the capacitance, as shown by EIS measurements, by increasing the diameter of the semicircle part of the response and increasing the Z’’ values at medium frequencies respectively. These initial layers, used to modify the electrode surface, appear to have blocking properties. This is due to the insulating properties of the protein layer [27,28,29,30]. As the surface is exposed to increasing concentrations of plasma, the accessibility of the Au surface to the redox probe becomes increasingly limited, leading to lower peak currents. 

Additionally, when dealing with electrochemical immunosensors, it is generally anticipated that the formation of an immunocomplex on the antibody-coated surface upon exposure to antigen, a further decrease in current should be observed. These characteristics have been observed for immunosensors in the majority of studies [27,28,29,30]. The EIS results further verify this observation as the Nyquist plot in Figure 4 shows that the charge transfer resistance increases with the exposure to increasing plasma concentrations. This phenomenon can be explained by taking the capacitance of the system into consideration. As reported previously [30], the total system capacitance for an Au/SAM/Antibody sensor involved three capacitive components in series is given by Equation (1) below:

(1) 1CT=1CSAM+1Cab+1Cdl

where *C_T_* is the total system capacitance, *C_SAM_* is the capacitance of the SAM layer, *C_ab_* is the capacitance of the biolayer and *C_dl_* is the double layer capacitance. The capacitance Equation (2) for a metal surface in contact with an electrolyte is:

(2) C=εε0Ad

where, for a bare electrode surface, *C* is the capacitance of the double layer (*C_dl_*), *ε* is the dielectric constant of the electrolyte (80 for water and much lower for organic materials such as proteins) and *ε*_0_ is the permittivity of the vacuum given to be 8.023 × 10^−12^. 

Naturally, for the bare Au surface the only capacitance present is *C_dl_* (or *C_stern_*) and therefore *C_T_* reduces to the Stern layer capacitance and its value ranges between 40−60 μF cm^−2^ [31]. In this case, where no SAM or biolayer is present, the only dielectric constant involved is that of H_2_O, which is given to be 80 [32,33] (note that dielectric constant values are free of units). Consideration of the voltammetric results suggests that the decrease in current upon formation of monolayers and bound antibody layers is attributed to the blocking properties of the layers. But where does this blocking property arise from? The study from Li et al. (2013) indicates that a range of dielectric constants are used in the literature for macromolecules but no set value has thus far been agreed upon [33]. They note that values of ε between 1 and 40 have been used in various studies but generally a value of ε = 4 is used. In any case it is evident that the dielectric constant of the proteins and other organic materials immobilised on the electrode surface is much smaller than that of H_2_O. As a result, as can be seen from Equation (2), the antibody layers have much smaller capacitances than the bare Au surface (and therefore much higher *R_ct_*), which is in full agreement with our experimental findings and those of others [31,34]. Upon addition of antigen, while the dielectric constant would in theory not change, the biolayer thickness increases as additional antibodies on the surface interact with newly arriving antigen and, as is predicted by the capacitance Equation (2), the total system capacitance should therefore decrease.

### 3.3. Detection of Plasma Immunoglobulin in AD and Control Subjects

**Figure 5:**
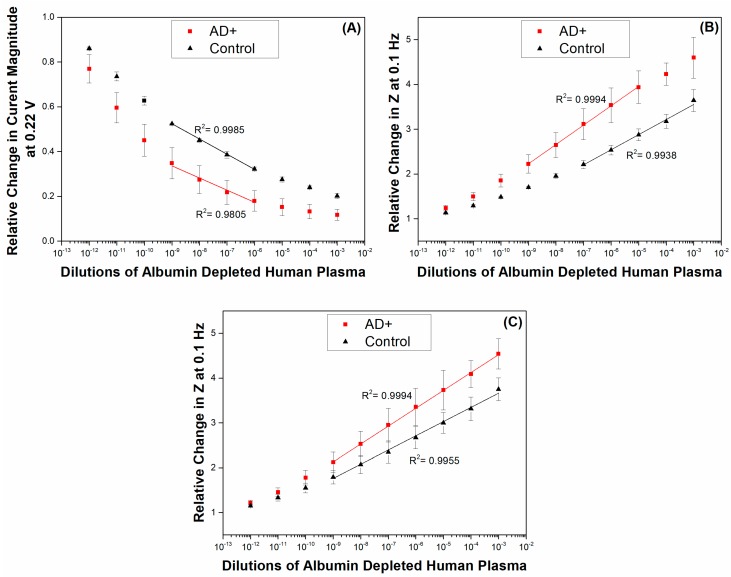
Logarithmic plots derived from (**A**) CV data showing the relative changes in current magnitudes at a bias potential of 0.22 V and (**B**) EIS data showing the relative changes in total impedance (Z) at 0.1 Hz, for AD (1) and control (2) cases against the baseline response (plain PBS) versus the range (10^−12^ to 10^−3^) of 10-fold dilutions of albumin depleted human plasma; (**C**) cumulative set for relative changes in total impedance (Z) at 0.1 Hz from AD cases (1 and 3) and control cases (2 and 4). Tested in 10 mM PBS containing 5 mM [Fe (CN)_6_]^4−/3−^ and 150 mM NaCl. Electrode surface area: 0.02 cm^2^. Error bars are sample standard deviations of measurements on *n* = 2 samples for (**A**,**B**) and 3 samples for (**C**). Z is given by the following equation $Z_{tot}=\sqrt{Z_{real}^{2}+Z_{imag}^{2}}$.

Figure 5A,B shows the calibration curves for AD (1) and control cases (2) derived from CV as current obtained at 0.22 V (Figure 3) and EIS as total impedance at 0.1 Hz (Figure 4) as well as the cumulative set of calibration curves using EIS data for AD (1 and 3) and control (2 and 4) cases. 

Due to the amount of manual work involved with the preparation of the sensing surface, it is likely that differences in the magnitude of the responses make it at time not feasible to directly compare the obtained values. It is therefore optimal to compare relative values—by the use of a blank-baseline sample before any plasma additions are carried out, it is possible to directly compare all data from the subsequent measurements with the data obtained for the baseline response in order to obtain relative increases in impedance. Additionally, by the use of a baseline response one may observe directly the surface drift which is common in all electrochemical measurements. In this manner, the drift response which is always highest at the beginning of the measurements is directly accounted for. For the analysis of the CV results in terms relative changes in current magnitude we selected the current values obtained at 0.22 V during the CV scans. In this manner, it is possible to directly compare the CV and EIS results for sensitivity using values obtained when the bias voltage was at the same value for both measurements. 

It is evident here that the control case displayed lower changes than the AD case, which is in line with our expectations. It is anticipated from the type of measurements being performed that changes in EIS display an increasing trend as the impedance of the system is becoming higher while changes in CV data are naturally of a negative nature, since the current magnitude is decreasing with increasing antigen concentrations. Figure 5A,B show that there were smaller induced changes in both the CV and EIS responses of the surface in the control (2) case in comparison to those observed in AD (1). The higher amounts of IgG present in the plasma of the AD (1) case correspond to higher amounts of IgG molecules being captured by the primary anti-human IgG antibody covered surface. We observe in Figure 5A that the sensing surface displays two linear ranges for each of the cases tested. The first linear range for both the AD (1) and control (2) cases appears between the dilutions of 10^−12^ and 10^−9^. The R^2^ values obtained from the analysis were 0.9874 for the AD (1) case and 0.9981 for the control (2) case. The second subsequent linear ranges for both samples appear between the dilutions of 10^−9^ and 10^−6^ with R^2^ values of 0.9805 and 0.9985 for the AD (1) and control (2), respectively. A plateau appears for the rest of the measurements suggesting that the sensor saturated or that the techniques’ sensitivity was limited beyond these ranges, suggesting therefore a limited sensitivity range of only 3 orders of magnitude. 

The EIS profiles suggest one linear range per case. For the AD (1) case, the best fit was obtained between 10^−9^ and 10^−5^ dilutions with a R^2^ = to 0.9994, which is very close to a straight line. A linear region is observed with R^2^ = 0.9938 for the control (2) case for plasma dilutions ranging from 10^−7^ to 10^−3^. As it is evident from this data, the AD (1) case showed higher responses than the control case and it appears that material is still being deposited in the control case at higher plasma concentrations, suggesting that the surface is not starting to saturate within the dilution ranges that we have investigated here. These results suggest that a higher amount of IgG molecules is present in AD (1) in comparison to the control case. At concentrations above 10^−5^ it is expected that the sensor surface has become saturated and a plateau is starting to appear in our AD (1) data set. The data obtained for the control subject indicate that a lower amount of IgG molecules is present in the control (2) sample and therefore the responses are much lower and linearity is only achieved at a concentration of 10^−7^ and above. 

Figure 5C presents the cumulative results from AD cases (1) and (3) and control cases (2) and (4). The addition and comparison with the extra AD and control samples shows a smaller difference between the AD and control cases. Additionally, the error bars obtained from AD (1) and (3) and control (2) and (4) samples appear larger than those obtained for cases (1) and (2) alone. The increased error bars appearing in Figure 5C can be attributed to the variation of IgG content among various cases. A number of reasons can induce such variations in IgG levels among different individuals which include differences in age, diet, polypharmacy and overall health. Infections and vaccinations occurring slightly before sample collection can further affect the IgG content. Therefore, further calibration with large number of samples for both control and AD patients will be needed. Nevertheless, this study showed the feasibility of a tool and device for providing information for rapid initial diagnosis of dementia.

## 4. Conclusions

In this study, the implementation of an electrochemical analysis for the detection of plasma immunoglobulins is reported. Differences in plasma immunoglobulins between control and dementia subjects were observed that the ELISA technique fails to demonstrate [23]. There are numerous problems inherent to immunoassays performed with human samples that need to be avoided when using bioassays for diagnostic purposes [35]. One of the major problems is the false-positive results due to interference as a result of heterophile antibodies and/or other interfering substances [36]. One way of minimising the interference, especially when detecting trace levels of disease specific biomarkers, is via selective antigen (biomarker) enrichment, to speed the binding kinetics with receptors and, thus, reduce signal interferences [37]. This novel assay has a number of advantages over the routine ELISA used to measure blood proteins, for example the immobilized biological material is present in intimate contact with a suitable transducer. Therefore, the biochemical signal is quickly converted into an electrical signal, the immobilization of biomolecular permits reuse of these molecules and allow simplification of the entire apparatus. The biological sensing element is present in a small area and is very sensitive, thus facilitating analysis of a substance in small quantities, and biosensors may be developed according to specific needs and can be highly specific. In addition, they have the advantage of being incorporated in small and portable equipment, thus having the potential to be used in routine clinical settings such as memory clinic services. This will facilitate the diagnosis of dementia in the community and enable earlier commencement of the currently available anti-dementia drugs. Furthermore, the proposed assay can find wider implications in primary care settings via development of ‘templates’ for certain medical conditions and enable more accurate diagnosis, monitoring of disease progression, and therapeutic outcomes. 

The modified gold electrodes were able to detect plasma Ig concentrations as low as 10^−9^ with linearity up to 10^−5^ dilution, placing the detection limit of the sensing interface in the range of pg mL^−1^ IgG. Our results suggest that the electrochemical technique will be more suitable for detecting plasma immunoglobin from samples with approximate protein content between 4 pg mL^−1^ and 4 ng mL^−1^ concentrations if it should be used in the detection of this AD biomarker, while higher IgG concentrations would inevitably saturate the surface. This *per sé* is an important issue, since it will enable the use of very minute amounts of blood sample to detect the differences between AD and control subjects, and this may be independent of the underlying anti-dementia therapy AD subjects had. Our latest findings are supported by newly recruited AD and control cohorts, where the AD drug-naive subjects were longitudinally followed at 1 month and 6 months after starting the anti-dementia treatments. The ELISA results confirm that the drug naive AD group had higher levels of plasma IgG that were normalized after 6 months following the anti-dementia treatment (EBM-L unpublished data). This suggests that the IgG differences between AD and control subjects exist prior to anti-dementia drug use and this difference disappears following anti-dementia treatment in AD as measured by routine ELISA assays. 

One of the limitations of the current study is the use of a polyclonal antibody against immunoglobulin that reacts with human IgA alpha chains, IgG gamma-chains and IgM-mu chains. Since the IgG is the most abundant immunoglobulin in human serum/plasma [accounting for 70–85% of the total immunoglobulin pool, with IgM and IgA accounting for <5% and 5–15% of the antibody pool, respectively [38]. We cannot exclude the possibility of cross reactivity with the minor representative immunoglobulin species in human plasma that the immunoprobe detects—IgA and IgM immunoglobulins. The next step in improving the biosensor assay is to utilise a monoclonal immunoprobe to minimise any cross-reactivities with other antibodies/immunoglobulins that may be present in the plasma of older people. 

Our novel immunoglobulin electrochemical assay, although not targeting a low expression plasma protein, shows an amplified sensitivity in detecting plasma immunoglobulins and differentiating between AD and healthy control subjects that we failed to achieve when using ELISA immunoassays. This finding is very encouraging and now needs to be explored further using different immunoprobes to determine the changes in other low-expressing blood proteins related to dementia hallmarks. If differences between AD and control subjects are confirmed, this will provide further support for electrochemical-based assays as good candidates to aid the further development of peripheral (blood) biomarkers for dementia.

## Supplementary Materials

The following are available online at http://www.mdpi.com/1424-8220/17/11/2464/s1, Figure S1: CVs (scan rate: 50 mV s^−1^) carried out for the rabbit anti-human IgG covered surface, baseline solution (plain PBS) and increasing plasma concentrations from 10^−12^ to 10^−3^ 10-fold dilutions of albumin depleted human plasma for the Control (2-a) case, Figure S2: EIS (respectively) measurements carried out at OCP of 0.22 mV for the rabbit anti-human IgG covered surface, baseline solution (plain PBS) and increasing plasma concentrations from 10^−12^ to 10^−3^ 10-fold dilutions of albumin depleted human plasma for the Control (2-a) case.
