# Supplementary material for: Electrochemical Detection of Plasma Immunoglobulin as a Biomarker for Alzheimer’s Disease
Source: Sensors (Basel). 2017 Oct 27;17(11):2464. doi: 10.3390/s17112464 (PMC5713623; doi:10.3390/s17112464)
Supplement: Supplementary file 1 [file sensors-17-02464-s001.pdf]

Article

Electrochemical Detection of Plasma Immunoglobulin as a Biomarker for Alzheimer's Disease

Goulielmos-Zois Garyfallou [1], Orlando Ketebu [1], Samet Şahin [1,2], Elizabeta B. Mukaetova-Ladinska [3], Michael Catt [4] and Eileen Hao Yu [1,*]

[1] School of Chemical Engineering and Advance Materials, Newcastle University, Newcastle upon Tyne NE1 7RU, UK; william.garyfallou@quantumdx.com (G.-Z.G.); orlandok2@yahoo.com (O.K.); sametsahin@me.com (S.Ş.)
[2] Department of Chemical and Process Engineering, Faculty of Engineering, Bilecik Şeyh Edebali University, 11230 Bilecik, Turkey
[3] Department of Neuroscience, Psychology and Behaviour, University of Leicester, University Road, Leicester LE1 7RH, UK; eml12@le.ac.uk
[4] Institute of Neuroscience, Faculty of Medical Sciences, Newcastle University, Newcastle upon Tyne NE2 4HH, UK; michael.catt@ncl.ac.uk
* Correspondence: eileen.yu@ncl.ac.uk

Supplementary Informatıon

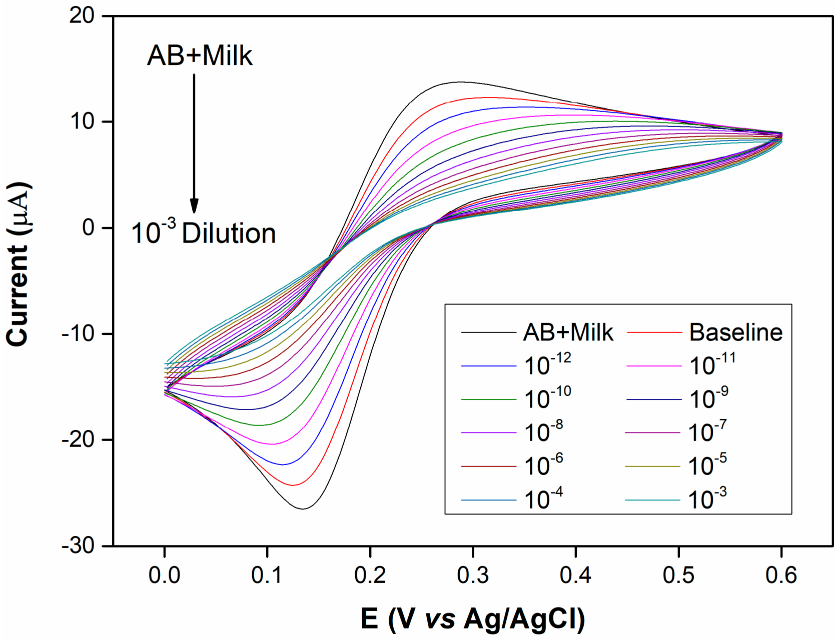

Figure S1. CVs (scan rate: 50 mV s^{-1}) carried out for the rabbit anti-human IgG covered surface, baseline solution (plain PBS) and increasing plasma concentrations from 10^{-12} to 10^{-3} 10-fold dilutions of albumin depleted human plasma for the Control (2-a) case. Tested in 10 mM PBS containing 5 mM $[Fe(CN)_6]^{4-/3-}$ and 150 mM NaCl. Electrode surface area: 0.02 cm^2.

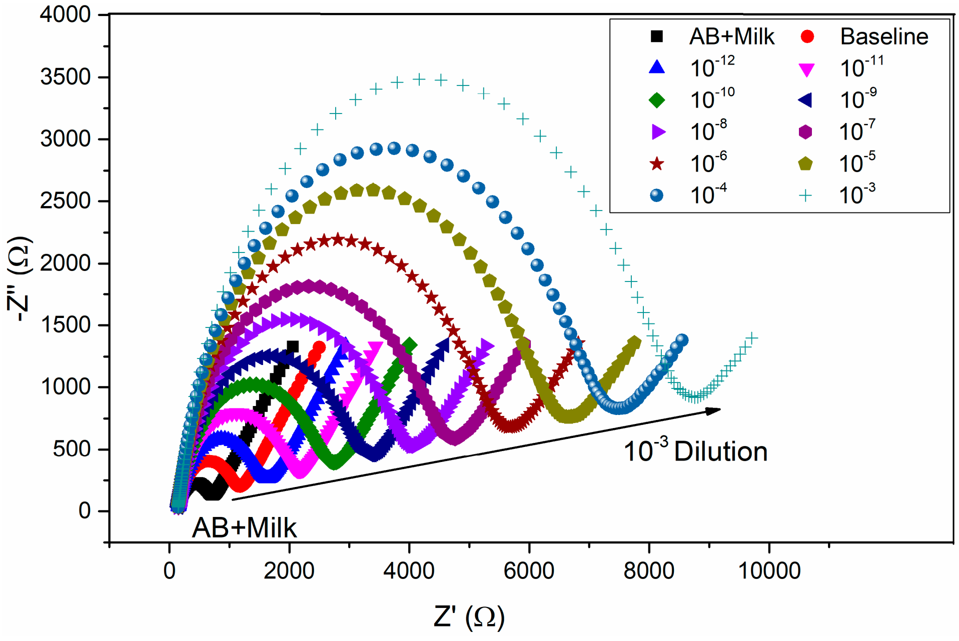

Figure S2. EIS (respectively) measurements carried out at OCP of 0.22 mV for the rabbit anti-human IgG covered surface, baseline solution (plain PBS) and increasing plasma concentrations from 10^{-12} to 10^{-3} 10-fold dilutions of albumin depleted human plasma for the Control (2-a) case. Inset figure is the electrical equivalent circuit which was used for data fitting and the solid lines represent fitted data. R_s is solution resistance, R_{cys} and C_{cys} are the resistance and capacitance of the cysteamine layer, R_{bl} is resistance of the biolayer and Q_{bl} a constant phase element representing the capacitance of the biolayer (n>0.7 for our data) and W is the Warburg impedance representing diffusion. EIS test parameters: AC amplitude: 10 mV, frequency range: 0.1 Hz – 10 kHz. Tested in 10 mM PBS containing 5 mM $[Fe(CN)_6]^{4-/3-}$ and 150 mM NaCl. Electrode surface area: 0.02 cm^2.
